# Technology as a Double-Edged Sword: Understanding Life Experiences and Coping With COVID-19 in India

**DOI:** 10.3389/fpsyg.2021.800827

**Published:** 2022-02-03

**Authors:** Girishwar Misra, Purnima Singh, Madhumita Ramakrishna, Pallavi Ramanathan

**Affiliations:** ^1^Department of Psychology, University of Delhi, New Delhi, India; ^2^Department of Humanities and Social Sciences, Indian Institute of Technology-Delhi, New Delhi, India; ^3^University of Queensland – IIT Delhi Academy of Research, New Delhi, India

**Keywords:** emotional well-being, digital technology, COVID-19, self-efficacy, resilience, work performance

## Abstract

The two waves of COVID-19 in India have had severe consequences for the lives of people. The Indian State-imposed various regulatory mechanisms like lockdowns, encouraged remote work, online teaching in academic institutions, and enforced adherence to the COVID protocols. The use of various technologies especially digital/online technologies not only helped to adapt to the “new normal” and cope with the disruptions in pursuing everyday activities but also to manage one’s well-being. However, the availability and accessibility of digital technologies to various sections of the population were not uniform. This paper reports a series of three studies examining the nature of pandemic stress, the impact of technology use on people’s emotional well-being during turbulent times, and the effects of technology use on psychological resources like resilience, self-efficacy, motivation to work, and emotional well-being. The differences in the residential background (Urban/Rural) and SES (Low/High) in the extent of the use of technology and strength of psychological resources were assessed. The findings indicated that the most common causes of concern included worrying about family, friends, partners, fears of getting and giving the viral infection to someone; frustration and or boredom; and changes in normal sleep patterns. It was noted that technology was a double-edged sword and created barriers as well as opportunities for the people. Also, self-efficacy mediated the relationship between the use of technology and emotional wellbeing. The results have policy implications for building resilient communities in the post COVID period.

## Introduction

While hearing the words COVID-19, myriad images of misery, pain, and panic come to our minds. A series of negative emotions is ignited and experienced, and memories of chaos and helplessness are activated. For most people, these emotions and memories are intensely frightening and deeply negative in nature. During the last 2 years, many countries across the continents were forced to impose lockdowns for months, enact unprecedented physical distancing, and ask to wear masks to contain the spread of COVID-19 and save human lives. Thus disruptions, discomforts, and affective disturbances were deliberately chosen so that breathing can be ensured. The impact of such extraordinary measures needs to be understood as they are bound to have delayed and long-term consequences for human development. Uncertainties about health, economic, social, and personal lives severely impact health, wellbeing, and productivity. Taken together they posed a variety of challenges for everybody irrespective of caste, creed, or religion (Sodi et al., 2021). The sudden changes and the fear of the unknown caused anxiety, fear, stress, and overwhelmed people. People have struggled with such a situation for more than a year. They had to deal with unmet expectations and unforeseen challenges, leaving everyone feeling extremely vulnerable. Given the situation, individuals devised their own ways of coping with the pandemic. In this context technology played a significant role, becoming essential for the completion of various tasks including maintenance of social relationships, and performing work-related tasks (Goldschmidt, 2020). The arrangement of life during the pandemic was characterized by social isolation and social distancing, and people tended to rely on technology to participate in professional activities, undertake various civilian assignments and tasks, and stay connected to the family, friends, and the world in general. In fact, technology seemed to help people maintain a grip on their social and emotional well-being. Technologies came to rescue and help navigate through the troubling events and happenings in the life world.

The two major waves of COVID-19 in India, one in 2020 and the other in 2021, have overawed the citizens in almost all respects-infrastructural, physical, mental, and emotional- as has occurred in many other parts of the world (Purkayastha et al., 2021). However, being the most populous democratic country in the world, India had to face a massive health emergency of enormous magnitude, leading to short-term as well as long-term impacts on the quality of personal, social, and community lives. During both the waves, the Indian Government adopted various regulatory mechanisms like imposing lockdowns, encouraging the practice of remote work, online educational instruction in academic institutions, and adherence to the COVID protocols in public places what has been called adherence to the “new normal.” During these times when direct face-to-face social interactions remained minimal, direct social interactions had increasingly been replaced by technology-enabled interactions as had happened in other parts of the world. With its great diversity, it is important to examine the extent to which the use of various technologies especially digital technologies have facilitated coping with this situation and enhanced emotional wellbeing. However, in the context of India, technology has emerged as a double-edged sword mainly because of the digital divide. While India has a major presence in the sphere of digital technologies at a global level and the volume of its growth has surpassed many countries, the availability, and accessibility in the different parts of the country and to various sections of the population are not equitable (APC News, 2021). According to a report by Digital in India (Global Digital Insights, 2021), the number of internet users was 624.0 million in January 2021. The internet penetration in India was 45.0 percent in January 2021. This report notes a vast increase in the total number of mobile connections is also seen with 1.10 billion mobile connections in January 2021 which is equivalent to 79.0 percent of the total population being mobile users. Despite this increasing expanse, it is also a reality that this technology benefited some people adapting to the challenges imposed by COVID-19 while a large section of the population, the marginalized, slum dwellers, poor, and those living in rural and remote areas could not avail the benefits of this vast expansion. It may be noted that while digital initiatives serve as the invisible thread holding the social fabric together, their efficacy in mitigating the stresses and fostering well-being depends on the availability and access to them. Against this backdrop this paper aimed at examining the perceptions of the use of technology and its impact on the emotional wellbeing of people from diverse segments of society. We argue that this relationship between technology use and well-being will be different for these segments. We also examine the differential use of technology as a barrier in some contexts and also provide opportunities in others.

### The COVID-19 Scenario in India

The first case of COVID-19 in India was reported on 30th January 2020. After it was declared as a pandemic by the WHO, on 19th March 2020, the Indian Prime Minister Narendra Modi addressed the nation on the issue of the pandemic calling for a self-imposed curfew on 22nd March, so as to prepare the citizens for the coming days (Jain et al., 2021). It has been noted that a strong national identity is useful as leverage when you are seeking to garner favors for sound public health, and precautionary actions for the benefit of the larger population (Van Bavel et al., 2020). Using this strategy, Modi imposed the first complete lockdown, which lasted 21 days. Overall, four lockdowns were imposed. Its frequency varied across different regions of India. Given the short gap after the announcement of the lockdown, panic and fear rose dramatically (Jha, 2020), people were stranded in different parts of the country with no mode of transportation to reach home, and the uncertainty of life and livelihood prevailed. This period also saw other socio-economic complexities such as lack of good healthcare infrastructure (Tejaswi, 2020), lack of resources to conduct education online (Sudevan, 2020), condition of the poor, labor migration (Rashid et al., 2020), uncertainties regarding the nature and impact of the virus, and concerns regarding the falling gross domestic product (OECD, 2020), among others.

The second wave of COVID-19 infections in India in March 2021 overwhelmed hospitals and urban and rural communities. According to a report by Centre for Science and Environment (CSE, 2021), the second wave in India, distressed the rural areas in terms of rate of infections, and lack of resources far more than the urban centers. The second wave of COVID-19 in India has had severe consequences in the form of spiraling cases, reduced supplies of medicines and essential treatments, and increased deaths particularly in the younger segment of the population (Asrani et al., 2021). The grim situation constrained the movements of people for a long time and left them desolate and apprehensive. We posit that the use of technology helped people negotiate life and overcome the situation in addition to other practices aimed at preventing disease and managing resources.

### Technology and Emotional Well-Being

The American Psychological Association (APA) has stated that spending entire weeks at home (with limited resource supply, reduced stimulation, and curtailed social contacts) can seriously damage people’s health and wellbeing, by intensifying the negative emotional states, such as fear, anxiety, depression, frustration, and/or irritability (APA, 2020). This has been demonstrated by a large number of studies examining the impact of COVID-19 (Cellini et al., 2020; Soraci et al., 2020; Riter et al., 2021; Singh and Quraishi, 2021; Zinchenko et al., 2021). Surbhi et al. (2021) noted that Indian undergraduate students particularly experienced heightened fear, anxiety, and stress and attempted to address these negative feeling states by practicing gratitude. Exclusive to COVID-19 is the use of digital communication technologies that have helped people cope with the pandemic, for example by reducing loneliness and isolation and increasing belongingness *via* social support (Gabbiadini et al., 2020). For the purpose of the present study, the use of technology includes various functions such as accessing technology for healthcare, for work/education, accessing resources such as groceries, etc., making digital payments, and staying in touch with friends and family. Thus, this variable encompasses not just the medium but also the access to technology. The use of technology, when conceptualized in this way, can be a boon, but may also be a stressor. Technology has proved to be beneficial during these times but limited access to certain groups has led to diminished impact, thus we also examine this relationship across various groups in order to understand any differences in the interaction of these variables. Further, the present series of studies examines not just the stressors experienced during COVID-19 times but also if the use of technology helped individuals’ resilience, motivation to work, and self-efficacy through fostering emotional well-being. Indeed, people stayed virtually connected with others through technology. Virtual conversations (e.g., phone calls, text messages, video chats, and interaction on social media) guaranteed social support, health care facilities, consultations, professional engagements, and education. Past research has highlighted two main social interaction processes (i) online sharing of emotions (Rimé et al., 2019), and (ii) online provision of social support (Herbert and Brunet, 2010). Sharing of emotions and availability of social support may be the key factors enhancing well-being.

Broadly, well-being is considered an individual’s appraisal of one’s quality of life (Diener, 1993). Emotional well-being is a key dimension of subjective well-being and explains the degree of the presence of positive feelings (Keyes, 2000). Theoretically, it is the balance of the feelings of positive and negative emotions. Keyes (2002) also suggests that it is measured by an individual’s response to structured scales measuring the presence of positive affect, the absence of negative affect, and how much a person is satisfied with life. Thus, it constitutes an important variable to explore in the context of COVID-19. It is essential to understand how a person’s quality of life, in terms of emotional well-being was impacted during COVID-19. Thus, this study took one step further and made an attempt to understand what might possibly alleviate the symptoms of reduced quality of life by exploring the role of technology. Hence, a series of studies have been designed to understand an individual’s experience of stress and to examine how the use of technology can influence emotional well-being. Such explorations will deepen our understanding of how these variables interact in real life and how they may inform social policy eventually. In total three studies were conducted. Study-1 assessed the pandemic stress. In particular, the various stressors experienced by people were identified. Study-2 analysed the factors influencing well-being and also the role of technology through in-depth interviews and Study-3 examined the use of technology and its relationship with emotional well-being. The role of self efficacy, resilience and motivation to work in mediating the effect of pandemic stress on emotional well-being was also examined.

## Study 1: Assessment of the Nature of Pandemic Stress

The Pandemic has created a considerable amount of uncertainty and stress among people (Sodi et al., 2021). At a global level stress was experienced by citizens in many nations due to the pandemic. Although both the developing and developed nations have been hit badly, the developing nations are hit harshly as they have inadequate health systems and are poorly prepared and this was true for India as well. When health, economic and other resources are inadequate the nature of stress experienced may be different. The first study attempted to measure the nature of pandemic stress in India. The specific behaviors and experiences of people were assessed.

### Method

A total of 328 participants (mean age = 29.32 years) participated in the study. The questionnaire was shared digitally, *via* Google forms platform to cover participants from across the country (pan- India). As the research focuses on studying the impact of access to technology - on life experiences and coping with COVID-19, data regarding the typology of residence (rural/town/metropolitan city/city) was collected in place of the geographical region. In this study, out of 328 participants – 11.58% (38 participants) were from a rural background, 17% (56 participants) from towns, 35.97% (118 participants) from metropolitan cities and 35.36% (116 participants) were from cities. Data collected included demographic details, perceptions of the role of technology in different areas of life (e.g., healthcare, accessing resources, digital payments, online education/work) and various psychological attributes (e.g., motivation to work, emotional well-being, resilience, and self-efficacy). The surveys were created in both Hindi and English to cover participants from different regions, genders, socio-economic levels and age groups. Participants provided details regarding their family structure, type of residence and socio-economic levels. The participants indicated their consent before commencement of their participation. Gender wise the data revealed that 55 percent of the participants were women. Analysis based on gender was abandoned as the mean scores obtained did not suggest substantial degree of gender differences. Additionally, 57 percent of the participants lived with their parents/spouse, 30 percent in a joint family and about 8 percent lived alone. There was an almost equal division amongst participants from lower and upper economic status levels with a 51.3 percent and 48.8 percent split, respectively. In this data, it was noted that 88 percent of the participants were from the urban sector, with 77 percent below the age of 36 years in age. The participants were residing in the different regions of India and contacted digitally during the peak of the pandemic in June 2021. The participants provided written consent and voluntarily participated in the study.

### Measures

This study used the Pandemic Stress Index (Harkness et al., 2020) to understand the most relevant concerns during the COVID-19 experience. The Pandemic Stress Index [PSI] (2021) was created to measure the stress and behavioral changes experienced by individuals during COVID-19. The Stress Index was initially created using a sample of Latino and sexual minority people at the University of Miami, by researchers at the Centre of Excellence for Health Disparities Research. It contains items that are self-administered, wherein participants respond to all items by checking the stressors experienced. It should be noted that the Pandemic Stress Index is not a scale, rather a checklist of specific experiences, which measures the psychological impact of COVID-19. Even though it was initially created for Latino and sexual minority men at the University of Miami, it has been widely used across different samples to measure stress experiences as the items in the checklist are too general to be applied in different contexts with varied samples. At present, this index is available in seven languages to cover wide geographical regions (Firkey et al., 2021; Hart and Han, 2021; Samuelson et al., 2021). The index studies three sections – behaviors during the COVID-19 pandemic, its impact on everyday life, and a checklist of the experiences during the pandemic. In this study, the section on experiences was used to map the extent of pandemic stress for participants. The checklist contains 19 items, wherein participants respond by checking all the stressors experienced. Examples of items include “Which of the following are you experiencing (or did you experience) during COVID-19 (Coronavirus)? (Check all that apply) - being diagnosed with COVID-19, fear of getting COVID-19, fear of giving COVID-19 to someone else, worrying about friends, family, partners, etc.” For this study, we used 18 items out of the 19 checklist items – as most respondents were uncomfortable responding to the item on “decrease/increase in sexual activity.”

### Procedure

The participants were requested to check all items and be reflective of their experiences. Percent frequency distribution was then, used to analyze the data and identify the pattern, as it is useful to observe the relative frequency of responses for the various components of the stress measure.

### Results

**FIGURE 1:**
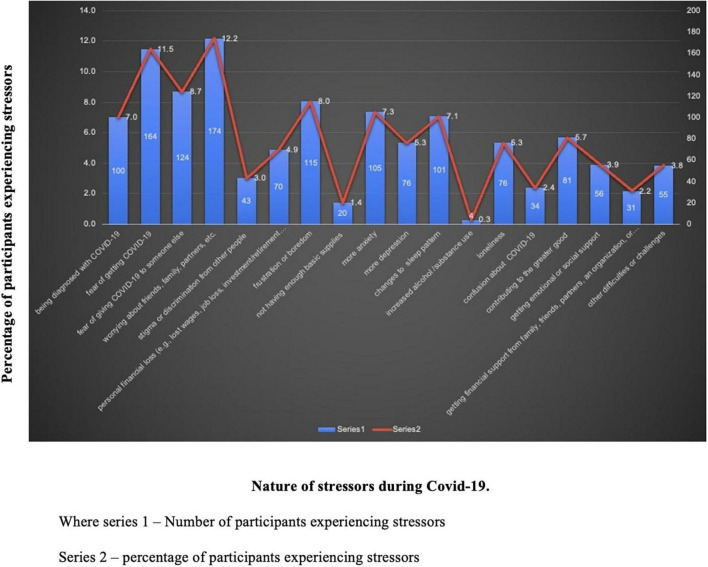
Percent frequencies of experiences of different stressors during COVID-19 pandemic.

The findings shown in Figure 1 indicated that the most common causes of concern included (in order): worrying about family, friends, partners, etc.; fear of getting COVID-19; fear of giving COVID-19 to someone; frustration and or boredom; and changes in normal sleep patterns.

The results showed that during the COVID-19 pandemic period people experienced a variety of stressors. Life has been taxing for most people, and concern for the well-being of family and friends emerged as a major stressor. During the period when the data were collected, the pandemic was at its peak and a significant number of people were critically ill and some even lost their lives. Fear of being infected with COVID-19 was an important concern and paranoia-like reaction about the disease was noted, from the responses in the Pandemic Stress Index. Self-concerns were also there because of the very nature of the pandemic. Concern for near and dear ones was a critical concern as there were incidents in which whole families and communities were affected. The lockdowns and long periods of being confined to the home and being far away from near and dear ones were other major stressors. Boredom, loneliness, and fear of being isolated from the community seemed to bother most people.

Overall, these results implicate that this pandemic period had proved to be very difficult for most of the participants and they experienced a large number of stressors that negatively affected the emotional well-being of people.

## Study 2: Impact of Technology on the Experience of COVID-19

Extending the preliminary exploration in Study-1, another study was designed to understand the experience of stress more closely. It focused on understanding the participants’ emotional well-being and related experiences during the pandemic. It also alluded to technology in an effort to understand the role played by it in pursuing the life activities and coping with challenges during the pandemic.

### Method

Semi structured, in-depth interviews were conducted (*n* = 25) from the Sikar district in Rajasthan, a state in Northern India. Out of the total number of in-depth interviews conducted there were 17 participants from a rural area and eight were an urban area. It was noted, that amongst the 25 participants, 12 were students, only four were homeworkers and the remaining were employed.

### Measures

The semi structured interviews dealt with the entire period of COVID-19. They focussed on impact of the pandemic on income, coping with stress, health, dealing with uncertainty and the overall effect of COVID-19 on everyday life. The interview also had items related to the role of technology during these times. The interviews were conducted telephonically, to observe the protocol of social distancing and an environment comfortable for the participant. The interviews were conducted after getting the verbal consent of the participants. The duration of interviews ranged from 30 to 75 min, with 45 min as the average length.

### Analysis

Post the data collection, transcription of the interviews was undertaken while taking care to note nuances in the voices of the participants. The data were then coded and analyzed following the procedure of thematic analysis (Braun and Clarke, 2006). Accordingly, the analysis included six steps. The first involves familiarizing oneself with the qualitative data, second – using line by line coding to generate initial codes, third – generating the themes, fourth – reviewing the themes, fifth – naming the themes and sixth – generating the report. While coding the data the research questions were used as a guide. As Braun and Clarke (2006) have noted a theme is a coherent and meaningful pattern in the data relevant to the research question. The analysis created a narrative involving the generated themes while supplementing them with excerpts from the data set. The purpose of the last step was to create a story that is comprehensible and scientific.

### Results

**FIGURE 2:**
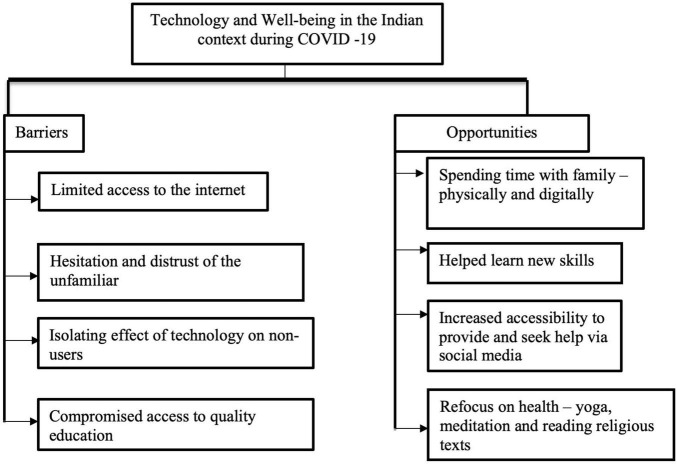
Themes emerging from interviews.

The themes emerging from the data obtained from interviews are presented in Figure 2.

It may be recalled that to control the magnitude of the COVID-19 pandemic, India had to undergo not one – but two phases of complete lockdowns (March 2020–June 2020 and April 2021–June 2021). Individuals across rural and urban landscapes had to adapt overnight to unfamiliar technologies given the stringent rules. It was found that contextual factors such as lack of a smartphones caused significant obstacles, including but not restricted to – being able to register for vaccination, access resources through online shopping sites and using applications for digital payment. Even though the number of India’s internet users increased by 47 million or 8.2 percent, the internet access remains restricted at 45 percent. To provide a more realistic assessment of the digital divide it may be noted that the number of mobile users in India stands at 1.10 billion, while the number of internet users is 624 million (Digital in India, 2021). These statistics provide a necessary context for the sub-themes, which is supplemented by the following excerpt:

“… *when the vaccination drive was happening, they (government) should have noted who has an android phone or not. For no reason, even during the registration for a vaccination, we faced obstacles. So, if one does not have an android phone, can they not get vaccinated? Now the government has understood, and made it offline. Initially it was a real struggle.*”

#### Barriers

##### Limited Access to the Internet

This theme emerged as a prominent concern. Irrespective of the professional status of the participants, limited network connectivity caused recurring interruption impeding their ability to cope with job requirements adequately. For example, a student shared his family’s woes in accessing teaching materials (“*my brother has to go to a field at a distance, to download the YouTube/video link, and he then comes home and studies it. So, in this sense, it has been inconvenient*”) while another working professional explained how even during COVID-19, she had to think differently to meet the job requirements – as most of her students did not have access to internet (“*First of all, the internet does not work. Secondly 60–70 percent of the people do not have android phones. We were very concerned for such students, and even tried to go to their homes, to keep their learning process intact*”). This concern reflected itself in other domains including digital payments (“*I do not struggle in understanding the process of digital payment, but sometimes the payment does not go through, as the server is down. So, when the payment fails, and charges are levied, it creates a problem then*”).

##### Compromised Access to Learning Opportunities

Related to the previous theme, the lack of stable and reliable internet connectivity has plagued both the parents as well as students. Many college students experienced heightened anxiety and negative mood state due to the high degree of uncertainty prevailing in the conduct of online education. Internet issues often led to irregular classes. As one student explained: “*there was a serious problem with the internet, due to which classes would not happen. We used to try 3–4 times, and then feel so annoyed, that we used to leave it. This affected our learning*.” The parents were additionally concerned, across both the rural as well as urban settings, on the quality of academic transaction during the span of over 2 years (“*I hear other parents also commenting that education has essentially stopped in the last two years. They say classes may be happening online. But what really is happening? Also, so many parents are uneducated. They are not even able to assess if their child is studying*”).

##### Hesitation and Distrust of the Unfamiliar

This theme was highlighted by several participants particularly among the elderly from the rural sector. Younger participants from urban areas displayed openness in adapting to newer ways of consulting doctors, and making digital payments etc. (“*there is a need to be more aware and friendly toward online consultation. There is limited awareness, in a small village such as Sikar. Like the younger generation is so familiar with all this*”). Older men from villages sometimes contrasted it to the “normal Indian way” of functioning, and explained their hesitation in shifting to online payment (“*they asked to pay online. I told them, what if my money goes into the wrong account, I cannot bring my money back.” “I am an ‘offline’ person*”) and for mobile-health consultations (“*one should sit in front of the doctor and take medicines, as they read the face and body structure, and then prescribe medicines. In the previous generations, this used to be the traditional practice, but it is very relevant in present time as well. Online consultations will take time to be adopted*”).

##### Alienation and Helplessness Among the Non-users

This theme is an important one and drew attention to those who were not employed, living alone or belonging to the group of elderly citizens, especially in the rural setting. A young student noticed how the pandemic was experienced as “more difficult” by her grandparents. She stated: “*It was quite difficult for them, as all the grandchildren are students, who are home and were involved in their studies. And their sons, who are working were also occupied online. So, it was quite difficult for them to pass time, even though it was a difficult time for everyone*.” A 55-year-old head instructor of a school noted that perhaps smartphones were the very reason why intense loneliness was experienced during the series of lockdowns (“*since android phones have come, man has become alone*…*if you give someone any work*…. *They say hmm – meaning he is so addicted he cannot see any other world around him*”).

#### Opportunities

##### Spending Time With Family

The idea that COVID-19 brought family members closer to each other was resonated by participants across ages and geographical expanses. This was especially highlighted by those in employment, and with children (“*First of all, because of COVID I got time at home – to spend with my children and family*” and “*In many ways, COVID brought my family back together*”). Others also highlighted how COVID brought back “good habits” amongst family members – “*A bit of routine came back in life with eating healthy, unlike sleeping late at night missing breakfast etc. We stopped running after money*.” It bridged the gap for those who were distanced physically, including encouraging some to switch to smartphones to communicate *via* video calls.

##### Helped Learn New Skills

This theme highlights the ways in which people sought self-improvement. The experiences shared by the participants showed nuances in terms of gender and geographical variation. Men in the villages used the internet to teach themselves to make bird feeders out of plastic bottles, as well as researching means of improving their crop (“*Some crops in the field had issues, they were not growing properly, leaves were dry and yellowing – my father researched and repaired the problems*”). Women in the village taught themselves some new recipes, while some in the city learned to drive, to encourage self-dependence (“*Learning driving has been a big achievement for me during these COVID times, because it is a necessary skill to know*”).

##### Increased Accessibility to Provide and Seek Help *via* Social Media

This theme highlighted an important contextual factor, especially in the rural sectors – the tradition and practice of serving and helping others (*Seva*). Many felt distressed and restricted because of COVID restrictions to help their community (“I*n my village, if anyone falls sick – I always used to try to help. Now there is a fear, because of which I have not been able to help. This is a big problem for me, and a source of pain*”). However, most participants lauded the role of social media in making help accessible nationally, irrespective of location (“*the help that people could access immediately was because of mobiles and internet. If someone lived in Sikar, people from Jaipur or other places could immediately tell where he could seek help*”).

##### Restoring the Focus on Health: Yoga, Meditation, and Reading

Some participants found alternative and socio-culturally relevant means of coping and restoring their emotional well-being. Given the Indian traditional practice of Yoga and Pranayama (breath regulation exercises), the findings indicated age as a non-criterion for such means of coping (“*I used exercise and yoga to cope mainly, because not only is yoga useful for weight loss – it also creates new energy within you. I also practiced pranayama.*”). Having said that, some participants shared that lockdown provided an opportunity to read inspirational and or religious books to cope with uncertainty. Findings indicated that younger participants leaned toward autobiographies and self-help books for inspiration, while older participants found solace in scriptures and religious texts.

### Conclusion

A detailed understanding of participants’ life experiences and well-being during the pandemic was gathered through this study. The thematic analysis of obtained data also showed evidence of the operation of certain key psychological variables. Themes such as “spending time with family,” “helping learn new skills,” and “refocusing on health” all indicated a strong sense of resilience and self-efficacy. Windle (2010) explains that “resilience is the process of effectively negotiating, adapting to, or managing significant sources of stress or trauma. Assets and resources within the individual, their life and environment, facilitate this capacity for adaptation and ‘bouncing back’ in the face of adversity” (pp, 12). In this way then, participants’ actions as described in the above-mentioned themes indicate a strong sense of resilience. Self-efficacy is an individual’s belief in their own capacity to perform behaviors necessary for specific outcomes (Bandura, 1977a). This too is explained by the narratives emerging from the interviews, where participants expressed an eagerness to learn new skills or work toward improving their circumstances. Further, participants discussed technology in detail as both an asset and a stressor in their work/education, highlighting the need to examine these variables closely. Thus, a third study was designed keeping these factors in mind, exploring specifically the relationship among variables such as the use of technology, motivation to work, self-efficacy, resilience, and emotional well-being.

## Study-3: Technology and Well-Being

This study examined the role of technology in shaping the well-being of the people during COVID-19 in greater detail, building upon the findings from the previous studies. The impact of COVID-19 on motivation to work, self-efficacy, and resilience was also explored. Additionally, the influence of the pandemic on perceptions of self-efficacy and whether such perceptions of self-efficacy mediated the relationship of technology use and well-being was also examined. For the purpose of this study, motivation to work was defined as participants’ desire to complete their work/education related assignments and tasks. We propose that higher work motivation (intrinsic) is linked to emotional well-being, as low work motivation has been linked with negative affect, depression, and anxiety (e.g., Lu, 1999). Björklund et al. (2013) also demonstrated that in organizations, employees with decreased work motivation had greater chance of experiencing exhaustion and depression in the future. These findings substantiate the need to include motivation to work as a key variable in the examination of emotional well-being, particularly in the context of COVID-19. Self-efficacy refers to a sense of confidence in an individual’s own capacity to perform tasks and is also related to the level of motivation (Bandura, 1977b). Schwarzer and Aristi (1997) explained general self-efficacy as referring to an individual’s overall self-confidence in dealing with the challenges of different environmental contexts or burgeoning issues (Xiong et al., 2020). It can reflect behaviors and actions across different contexts. Self-efficacy is an important aspect of assessing the quality of life and happiness of participants. Dogan et al. (2013) demonstrated that well-being and self-efficacy have a positive and significant relationship and together influence happiness. Thus, it was crucial to include in this study.

The concept of resilience has been defined in many ways and applied to many fields over the years. For the present study resilience was operationalized as a process involving a way of “effectively negotiating, adapting to, or managing significant sources of stress or trauma” (Windle, 2010, p.1). Over time, research on resilience has moved from deficit models addressing psychopathology toward models more focused on growth and healthy development. The present study explored resilience as both addressing a deficit and also healthy development. We propose that increased levels of resilience would predict increased emotional well-being. Keeping in mind the context of the pandemic, we included factors such as self-efficacy and motivation to work as additional predictors for emotional well-being. In this way resilience is understood not only as a precursor to emotional well-being, but also as a buffer from the increasingly uncertain and anxious world of the pandemic. Thus, this study addressed the experience of the emotional well-being of people during the pandemic through a detailed exploration of the extent of usage and access to technology, their motivation to work, level of resilience, and self-efficacy. A multi-pronged statistical analysis was designed to address this complex relationship.

### Hypotheses

**H1:** Residential background (Urban/Rural) and SES (Low/High) would yield significant differences in the extent of the use of technology, motivation to work, emotional well-being, resilience, and self-efficacy.
**H2:** Higher scores on the use of technology would be associated with higher scores on motivation to work, emotional well-being, resilience, and self-efficacy.
**H3:** Higher scores on motivation to work would be associated with higher scores on emotional well-being, resilience, and self-efficacy.
**H4:** Higher scores on emotional well-being would be associated with higher scores on resilience and self-efficacy.
**H5:** Higher scores on resilience would be associated with higher scores on self-efficacy.
**H6:** Use of technology, motivation to work, emotional well-being, resilience, self-efficacy, SES, and age would significantly predict levels of emotional well-being.
**H7:** Increased use of technology would predict an increase in emotional well-being (direct effect).
**H8:** Self-efficacy would mediate the effect of the use of technology on emotional well-being.

### Method

A total of 328 participants (mean age = 29.32 years) participated in the study.

### Measures

#### Impact of Technology

It was measured using a 16-item scale constructed by the authors. The items were judged with the help of experts. The items of this measure addressed the impact of technology on the following dimensions - work/education, online payment, mobile-health (m health) consultations, accessing resources and social/virtual life. This section assessed the participant’s access to technology and its role in coping with the pandemic. The items of the measure were rated on a scale ranging from 1 (strongly disagree) to 5 (strongly agree). This scale included items such as “Due to limited access to internet and latest technology, I have struggled to be productive at work/college” and “I experienced significant difficulty in adapting to new means of online payment.” The value of Cronbach’s α for this measure was found to be 0.77 and the Split-half reliability of the scale was 0.88.

#### Motivation to Work

It was measured using a scale constructed by the authors. The items were constructed and finalized with the help of experts. The seven items were rated on a scale ranging from 1 (strongly disagree) to 5 (strongly agree). This scale included items such as “The time I spend on family responsibilities predominantly interferes with my work responsibilities” and “It is not always easy for me to perform tasks on time.” Cronbach’s α for this measure was estimated to be 0.82 and the Split –half reliability of the scale was 0.85.

#### Emotional Well-Being

It was measured using the Mental Health Continuum Long Form (MHC-LF; Keyes, 2002, 2005). Five items of the six-item emotional well-being scale (EWB1) were used which included items such as “I felt cheerful most of the time” and “I was extremely happy most of the time.” The sixth item “I felt full of life, most of the time,” was not included as the item was not clear to respondents. The five items were rated on a scale ranging from 1 (strongly disagree) to 5 (strongly agree). Cronbach’s α for these five items was found to be 0.85 and the Split-half reliability of the scale was 0.86.

#### Resilience

It was measured using the Resilience Appraisal Scale (Johnson et al., 2010). For this study, all 12 items of the scale were used which measured the participant’s capacity to appraise their ability to solve problems, gain social support and cope with their emotions. The items were rated on a scale ranging from 1 (strongly disagree) to 5 (strongly agree). The scale included items such as “I can usually find a way of overcoming problems” and “In difficult situations, I can manage my emotions.” Cronbach’s α for this measure was found to be 0.90 and Split-half reliability of the scale was 0.94.

#### Self-Efficacy

It was measured using General Self efficacy scale by Schwarzer and Jerusalem (1995). All the 10 items of this scale were used which included items such as “Thanks to my resourcefulness, I know how to handle unforeseen situations” and “If I am in trouble, I can usually think of a solution.” The 10 items were rated on a scale ranging from 1 strongly disagree) to 5 (strongly agree). Cronbach’s α for this measure was found to be 0.88 and Split-half reliability of the scale was 0.92.

The reliability coefficients are indicated in Table 1.

**TABLE 1:** Split-half and Cronbach alpha values for measures used in study 3.

Measures	Guttman’s Lambda 4	Cronbach alpha
Impact of technology	0.88	0.77
Motivation to work	0.85	0.82
Emotional well-being	0.86	0.85
Resilience	0.94	0.9
Self-efficacy	0.92	0.88

### Analysis and Results

The analysis was done using R Studio Version 1.2.1335. The reverse scoring was done where required, and scaling was also done to ensure equivalence. The descriptive statistics were calculated and are demonstrated in Table 2. The table indicates the means, standard deviations, and correlations of the variables relevant to this study.

**TABLE 2:** Means, standard deviations, and correlations for variables.

Variables	n	Mean	SD	1	2	3	4	5
Age	328	29.32	13.36					
Gender: male	148	29.69	13.39					
Female	180	29.01	13.36					
SES: lower	168							
Upper	160							
Impact of technology	328	52.61	8.99	–				
Work motivation and performance	328	22.33	5.84	0.54***	–			
Emotional well-being	328	15.38	4.44	0.31***	–0.05	–		
Resilience	328	43.05	8.61	0.54***	0.18**	0.50***	–	
Self-efficacy	328	35.03	7.15	0.48***	0.16**	0.47***	0.71***	–

**p < 0.01; ***p < 0.001; Numbers 1–5 in the title row indicate the variables under consideration and are annotated as such for describing the correlations.

Correlations among the variables showed strong relationships, leading to acceptance of first four hypotheses. However, emotional well-being was negatively related to motivation to work (leading to partial rejection of Hypothesis-2), indicating that an increase in motivation to work was associated with a decrease in the level of emotional well-being. The Welch’s t-test was performed on the five variables under consideration, this resulted in adjusted values for df. These variables were grouped by both socioeconomic status (Table 3) and place of residence (Table 4). Overall, we may note that socio-economic status emerged to be important for understanding life experiences during the period of pandemic. The place of residence was also relevant particularly in understanding the use, access, and impact of technology during the pandemic. Thus Hypothesis 1 could be partially accepted. These results complement those discussed in studies 1 and 2.

**TABLE 3:** Mean comparison of scores of low and high SES groups on various measures.

Measures	Socioeconomic status (SES)				*t*(df)	*p*
	Lower		Upper			
	*M*	SD	*M*	SD		
Impact of technology	50.97	10.32	54.32	6.96	3.46(294.06)	0.000
Motivation to work	22.48	5.94	22.17	5.76	0.47(325.89)	0.635
Emotional well-being	15.03	4.43	15.74	4.43	1.44(325.21)	0.15
Resilience	40.16	9.46	46.09	6.36	6.67(293.76)	0.000
Self-efficacy	33.69	7.43	36.43	6.57	3.54(324.24)	0.000

*Welch’s T test was used for calculations.*

**TABLE 4:** Mean comparison of scores of rural and urban groups on various measures.

Measures	Place of Residence				*t*(df)	*p*
	Rural		Urban			
	*M*	SD	*M*	SD		
Impact of technology	55.26	5.97	52.26	9.26	2.70 (63.25)	0.008
Motivation to work	23.87	5.29	22.12	5.90	1.88 (49.84)	0.065
Emotional well-being	15.37	3.91	15.38	4.51	0.01 (50.86)	0.991
Resilience	41.26	6.34	43.29	8.85	1.76 (57.85)	0.083
Self-efficacy	34.50	5.77	35.10	7.32	0.579 (53.90)	0.565

*Welch’s T test was used for calculations.*

Further, a standard multiple regression was performed to obtain a clearer understanding of these relationships. It may be noted that as place of residence was significant only with variable considering the impact of technology, and barely so with work motivation, only socioeconomic status was considered for the purpose of the regression model. For the multiple regression model, impact of technology, motivation to work, resilience, self-efficacy, age, and socioeconomic status were regressed upon emotional well-being in order to understand the relationships underlying the individual’s life experiences during COVID-19. A significant regression equation was found [F(6, 321) = 27.96, p: 0.001), with an R^2^ of 0.3432 (Table 5), leading to the partial acceptance of Hypothesis-6. The multiple regression analysis meets all the model assumptions. However, the coefficients for motivation to work and socioeconomic status were negative, indicating that as their values rise, levels of emotional well-being diminish. This was supported by the previous study where participants had stated about many negative experiences regarding work during the pandemic, particularly due to lack of access and resources, and familial pressures. In this sense work became associated with stress. Although the impact of technology predicts an increase in emotional well-being, the negative relationship with motivation to work indicates that access to technology is clearly not the only facet of importance when understanding work motivations and experiences during the pandemic. Further, since the high-level SES was also negative, we can say that the model was found to hold true more so for individuals of lower SES. This seems consistent both theoretically and empirically, as evidenced in the previous study. To conclude, we may reasonably say that the impact of technology, self-efficacy, resilience, and age significantly predicted the level of emotional well-being, however low levels of motivation to work predicted emotional well-being rather than high level. Further, this effect was more prominent for the participants belonging to a lower socioeconomic status.

**TABLE 5:** Summary of regression analysis predicting emotional well-being.

	β	SE β	*t*	*p*
Impact of technology	0.221	0.066	3.364	<0.001
Self-efficacy	0.162	0.066	2.447	<0.05
Age	0.133	0.048	2.754	<0.01
Work motivation and performance	–0.241	0.055	–4.406	<0.001
Resilience	0.338	0.071	4.77	<0.001
SES upper	–0.278	0.099	–2.836	<0.01
Constant	0.136	0.066	2.065	<0.5
Observations			328	
R^2^			0.343	
Adjusted R^2^			0.331	
F Statistic			27.959 (df = 6; 321)	

**FIGURE 3:**
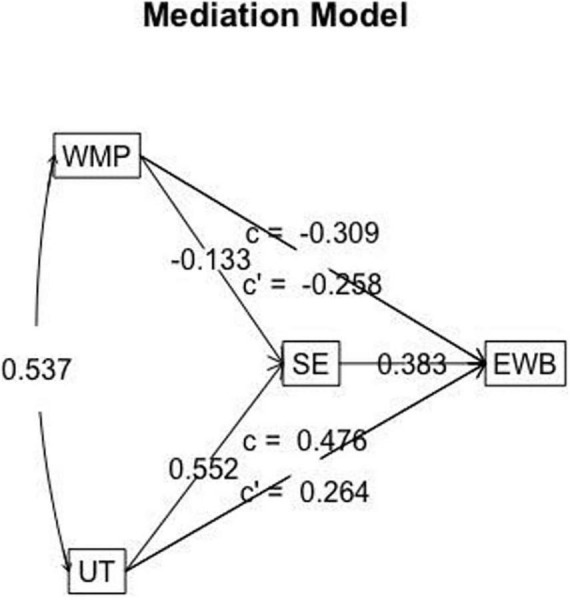
Mediation model predicting emotional well-being.

Does self-efficacy mediate the relationship between the Impact of Technology and Emotional well-being? Can motivation to work predict the impact of technology on emotional well-being? The analysis explored motivation to work as a predictor of the impact of technology, which in turn predicted emotional well-being, as mediated by self- efficacy. Figure 3 displays the mediation model analysis and the effect estimates, and Table 6 displays the fitness indices obtained through the mediation analysis. The obtained model indicated a moderate fit (Hu and Bentler, 1999; Kline, 2005), as despite the χ^2^ being significant, and χ^2^/df more than 3, other indications are within the range. It is noted that the χ^2^ statistic is very sensitive to sample size and is no longer relied upon as a basis for acceptance or rejection (Schermelleh-Engel et al., 2003; Vandenberg, 2006). Thus, the model appears to be a good fit. However, as also indicated by the correlations regression analyses, here too, motivation to work negatively predicted emotional well-being. This is termed as inconsistent mediation (MacKinnon et al., 2007), but since the magnitude of the direct effect is larger than the indirect effect, it may be said that an increase in motivation to work will lead to a decrease in emotional well-being. However, higher impact of technology will predict higher levels of emotional well-being when mediated by self- efficacy. Thus, it was found that increased impact of technology led to increase in levels of emotional well-being (direct effect), and self-efficacy mediated the effect of impact of technology on the levels of emotional well-being (indirect effect).

**TABLE 6:** Fitness indices for mediation model.

Indices	Obtained value	Recommended value
χ^2^/df	5.381	3
*p*	0.02	*p* > 0.05
CFI	0.986	>0.90
TLI	0.913	≥ 0.95
NNFI	0.913	≥0.95
RMSEA	0.116	<0.08
SRMR	0.033	<0.08
GFI	0.992	≥0.95
AGFI	0.916	≥0.90

*According to Hu and Bentler (1999); and Kline (2005).*

## General Discussion

COVID-19 has been an unprecedented experience and people have experienced myriad stressors during this time; even today in many parts of the world, the onset of another wave has been noted. India being one of the most populous countries has grappled with this pandemic in its own way. Indians suffer significantly from infectious diseases on account of a complex interplay of demographic, environmental, and socio-economic factors (Dikid et al., 2013). This can also be seen in the case of COVID-19.

This research attempted to understand the nature of pandemic stress, the impact of technology use on people’s emotional well-being during turbulent times, and the effects of technology use on psychological resources like resilience, self-efficacy, motivation to work, and emotional well-being. Study 1 explored pandemic related stress in detail. The findings indicated that the most common causes of concern were worrying about family, friends, partners, etc., fear of getting COVID-19 and fear of giving COVID-19 to someone else. Study 2 explored the experiences of the pandemic stress more closely – it highlighted how “spending time with family,” “helping learn new skills,” and “refocusing on health” helped build a strong sense of resilience and self-efficacy. The final study (Study 3) highlighted that an increased impact of technology led to increase in levels of emotional well-being, while self-efficacy mediated the effect of impact of technology on the levels of emotional well-being. Most studies of technology use in the pandemic have demonstrated negative consequences on well-being (e.g., Aziz Rahman et al., 2020; Rajkumar, 2020; Pandya and Lodha, 2021). However, the present study is unique in that it demonstrates a positive relationship between the impact of technology and well-being.

Study 1 explored the level of pandemic stress experienced by participants, while also identifying the central concerns that people had during the peak of the pandemic. The Pandemic Stress Index (Harkness et al., 2020) was used as a checklist to measure the psychological impact of the pandemic on the Indian population. Through the analysis of this checklist, concern and worry for family and friends emerged as the primary stressor. India presented a grim picture in terms of health statistics between March 2020 to August 2021. According to the John Hopkins Coronavirus Resource Centre (Covid-19 map, 2021), India has had 33,953,475 citizens infected and witnessed the death of 4, 51,000. At present (October 2021), the COVID-19 positivity rate stands at 2.45%. A leading national daily (The Hindu, 2021) elaborated on the second wave being particularly difficult for those in rural expanses, as limited inoculation sites made vaccine availability difficult leaving a sizable segment of the population vulnerable. It is natural then, that concern for loved ones would be central in the thoughts of people, as India and Indians experienced so much loss and death.

Given the expanded temporal horizon of health emergency of unspecific nature, there were wide-ranging consequences for mental health and wellbeing. Study 2 explored the impact of these stressors in greater detail. Semi-structured interviews were conducted to understand these experiences, while alluding to the role technology played in coping with this unprecedented times. Through thematic analysis, the findings indicated that participants in the rural sector struggled to trust technology, which consequently acted as a barrier in accessing healthcare and education. However, the narratives emerging from the interviews also demonstrated the participants’ eagerness to learn new skills or work toward improving their circumstances across rural and urban sectors. Additionally, participants discussed technology as both an asset and a stressor in their work/education in detail, highlighting the need to examine these variables closely. These results were substantiated through a study by Venkatesh et al. (2021) wherein they found that Indian citizens used the time of the pandemic to build on their feelings of gratitude, personal strength, and professional commitment.

Technology was used during this time to allay fears and anxieties. For many people this was a new way of negotiating life demands and meeting the daily needs. This was perhaps brought about only because of the pandemic (De’ et al., 2020). The results of Study-3 showed that rural people were more significantly impacted by technology as compared to urban. Rural India has had a 13 per cent increase in Internet users in 2020, according to a report released by Internet and Mobile Association of India (IAMAI) and consulting firm Kantar (2021). The results of the present study, if examined in the context of the mobile technology expanse in the rural parts of the country, suggest that perhaps it was used for many other purposes during this time. Study 3 found the impact of technology was significant in many ways, particularly the way it is related to emotional well-being. It predicted emotional well-being both through regression (along with other variables) as well as through the mediation of self-efficacy. Although the usual literature trends suggest that increased screen time can lead to decreased mental health and well-being (Paulich et al., 2021), the present study, through its exploration of the impact and access of technology demonstrated that for people of lower socio-economic status, technology had a positive impact on emotional well-being. Goldschmidt (2020) too explained how technology became an essential part of the life during pandemic, and was important to maintain the well-being of children in particular. Pandya and Lodha (2021) also hinted at the possibility that use of technology may not interfere with well-being during times of social distancing; something that the present study has demonstrated.

The present results showed that many kinds of stressors such as anxiety about family, friends and self being infected, loneliness and boredom and unusual sleep patterns were experienced as major threats to wellbeing. The widespread experience of stressors has certainly made people miserable. Further, the ambiguity and indefinite nature of the disease condition and lack of information available enhanced the trauma. This period resulted in many kinds of resource-loss which may have contributed to the increased anxiety and tension. Two kinds of resource-loss was salient during this period: availability and access on account of restrictions imposed during the lockdown periods. In India, due to COVID-19, the uncontrollability, uncertainty, sudden changes and the fear of the unknown caused stress, fear and anxiety. This pushed for innovative thinking and out of the box solutions including creation of multiple vaccines and various testing facilities (Sodi et al., 2021).

We must acknowledge the rural-urban divide when it comes to technology access, more and more people are presently using it in rural areas. Compulsions to restrict movement and spatial confinement also facilitated the use of technology. It was a means for social connectedness, to relate to people, talk to them, and ask for help when needed. It was very helpful to access medical help and many medical professionals and hospitals started online consultations which facilitated medical help despite restrictions on movement. When access to medical facilities became problematic, tele-communication for medical consultations and delivery of various health facilities became critical; this helped people to carry on with their lives despite the stresses and strains all around. In many parts of the country during the Pandemic Wave-II oxygen/scarcity and lack of facilities, even essential drugs for the treatment of COVID-19 became a major concern. The multi-pronged efforts initiated at various levels helped to overcome this situation in due course.

Although our study was unique in many ways, certain limitations were noted. We developed two scales – impact of technology and work motivation and performance – for this study both having good reliability. More studies need to be conducted with these scales. These studies must also expand their reach to all sections of the society. While the present study attempted to capture differences between rural and urban, socioeconomic status, as well as gender, however these variables require a closer examination. The need to understand and include gender differences in access to technology is acknowledged. The scope of the qualitative study may be increased in further studies but for the present study as the interviews were very detailed and very informative, it was found to be sufficient.

### The Lessons Learnt During Covid

The pandemic required most people to redesign the different segments of life - personal, societal, economic and political at the local, national, and global level. Despite recurring issues, the use of online mediums for work and education is the new normal. Travel has become less frequent, undertaken only when there is a dire need. Assembly elections took place during the pandemic, and the thickly populated states like Bihar and West Bengal witnessed considerable mass mobilization during the pandemic. It is commendable that they were successfully completed even though there were many restrictions. It was inspiring to see that many aspects of life were being restructured by people to allow not just for survival but to achieve a degree of functionality. What is interesting to see is that this enhanced the feelings of self-efficacy and gave many people a new-found confidence to adapt to a “new normal,” to continue work, and to live life well (Varshney, 2021). A stronger society emerged after the various onslaughts of the pandemic. This strength has emerged from collaborative efforts of all the stake holders’ i.e., Government, voluntary organizations, health practitioners and people in general. Many people employed self-focused auto regulatory coping strategies like monitoring own thoughts, actions and yogic and meditational practices. Completing the vaccination of a significant chunk of the population was no easy feat. The drive began in January 2021, and concerted efforts by the government and various stakeholders resulted in the administration of 95 crore COVID-19 vaccine doses by October, 2021. This is not a meager achievement.

Psychological preparedness was also critical in enhancing our understanding of the relationships hypothesized. It is central to the understanding of the impact of disasters as it involves an individual’s capacity to anticipate and monitor their response “readily” in face of an emergency (Reser and Morrissey, 2009). As indicated by Malkina-Pykh and Pykh (2013) preparedness involves factors such as outcome expectancy, risk perception, collective efficacy, self-efficacy, coping style, perceived responsibility, and access to resources. Given that India experienced two severe COVID waves, one can question if the population responded with better psychological health in the year 2021 – given the psychological preparedness to cope with a disaster. Is the capacity to monitor one’s resilience and efficacy (self and collective) contingent on previous recent experiences? Therefore, the latent impact of the temporal factors in conducting research on the psychological impact of COVID-19 is pivotal to consider. Both resilience and survival have helped in dealing with the situation. Our resilience as a community is high which is the outcome of coping skills, social support that promoted positive adaptations to the pandemic related crises. Our survival has empowered us, given us agency, and can be seen in high self-efficacy. The concept of community resilience is thus critical in this regard. It is understood as the ability of a community to use its assets to strengthen systems such that they can improve the community’s health across various dimensions (The Print, 2021). It enables a community to withstand, adapt to, and recover from difficulties (Patel et al., 2017). Despite restrictions on movements during COVID, community resilience played an important role for Indian society. In a collectivistic culture the social and cultural networks and practices of the community are a major resource which helps in coping and alleviation of both individual and community stress. Oftentimes the community came together to help people in need. Future research focusing on community resilience in collectivistic cultures will reveal valuable insights on well-being outcomes and technology in the context of COVID-19. It is important to focus on the positive as well as the negative aspects, and perhaps community resilience and community togetherness are the silver lining emerging from the life experiences of COVID-19.

## Data Availability Statement

The raw data supporting the conclusions of this article will be made available by the authors, without undue reservation.

## Ethics Statement

The studies involving human participants were reviewed and approved by the Ethics Committee at the Indian Institute of Technology, Delhi. The patients/participants provided their written informed consent to participate in this study.

## Author Contributions

GM, PS, and MR conceptualized and designed the study. PS and MR led the data collection. MR and PR performed the analysis and interpretation of the data. GM, PS, MR, and PR wrote the initial draft and revised the subsequent manuscripts. All authors read, revised, and approved the final manuscript.

## Conflict of Interest

The authors declare that the research was conducted in the absence of any commercial or financial relationships that could be construed as a potential conflict of interest.

## Publisher’s Note

All claims expressed in this article are solely those of the authors and do not necessarily represent those of their affiliated organizations, or those of the publisher, the editors and the reviewers. Any product that may be evaluated in this article, or claim that may be made by its manufacturer, is not guaranteed or endorsed by the publisher.
