# Supplementary material for: Technology as a Double-Edged Sword: Understanding Life Experiences and Coping With COVID-19 in India
Source: Front Psychol. 2022 Feb 3;12:800827. doi: 10.3389/fpsyg.2021.800827 (PMC8850397; doi:10.3389/fpsyg.2021.800827)
Supplement: Supplementary file 1 [file Data_Sheet_1.pdf]

APPENDIX

Questionnaire in English

Dear Participants,

We are working on a research project to understand the impact of covid on people living in various regions of the country. Your responses will help us to know how people dealt with the issues related to pandemic at an individual and collective level. There are no correct or incorrect answers. For us your responses are the right responses. You will not be asked for any identifying information (e.g., your name or address). Information is completely anonymous. We assure you of complete confidentiality. The survey will take no more than 20 minutes of your valuable time. Your participation in this survey is completely voluntary.

If you have any questions, please contact misragirishwar@gmail.com, p.singh@hss.iitd.ac.in or Madhumita.ramakrishna@uqidar.iitd.ac.in

Please give your consent to participate in this survey. Yes------------------- No------------------

This survey is divided into 3 sections.

Section 1

Kindly provide some information about yourself. This information provided by you will be used only for research purpose.

Age:_______________________________________

Gender: ____________________________________

Place of Residence: ☐Metropoliton City ☐City ☐Town ☐Rural

Marital Status: ☐Married ☐Unmarried ☐Divorced ☐Widowed

According to you your Socio-economic Level: ☐ Lower ☐ Lower Middle ☐Upper Middle ☐Upper

What is the structure of your family? Please tick the ones that apply to you.

a) I live alone.

b) I live with my parents and/or spouse.

c) I live in a joint-family.

d) I have young children at home who are also having their own online classes.

e) Other (please specify): _________

Which of the following are you experiencing (or did you experience) during COVID-19 (coronavirus)? (check all that apply). Please also rank them, giving rank 1 to the experience which was most dominant and then descending order.

__ being diagnosed with COVID-19

__ fear of getting COVID-19

__ fear of giving COVID-19 to someone else

__ worrying about friends, family, partners, etc.

__ stigma or discrimination from other people (e.g., people treating you differently because of your identity, having symptoms, or other factors related to COVID-19)

__ personal financial loss (e.g., lost wages, job loss, investment/retirement loss, travel-related cancelations)

__ frustration or boredom

__ not having enough basic supplies (e.g., food, water, medications, a place to stay)

__ more anxiety

__ more depression

__ more sleep, less sleep, or other changes to your normal sleep pattern

__ increased alcohol or other substance use

__ loneliness

__ confusion about what COVID-19 is, how to prevent it, or why social distancing/isolation/quarantines are needed

__ feeling that I was contributing to the greater good by preventing myself or others from getting COVID-19

__ getting emotional or social support from family, friends, partners, a counsellor, or someone else

__ getting financial support from family, friends, partners, an organization, or someone else

__ other difficulties or challenges

Section 2

In this section you are being asked questions related to various technologies that helped you to carry on your work during the pandemic times. Each statement corresponds with a 5-point rating scale (Strongly disagree, disagree, unsure, agree and strongly agree). If the statement applies to you, please tick the appropriate rating.

**S.no**		**Strongly Disagree**	**Disagree**	**Unsure**	**Agree**	**Strongly Agree**
1	It was easy for me to obtain the healthcare services I needed within the past 12 months	1	2	3	4	5
2	Information from the following sources or channels (school, work, hospitals, TV) regarding healthcare was readily available.	1	2	3	4	5
3	When needed, I could afford (financially) to access: physician, specialist, hospitals, medicines and medical equipment	1	2	3	4	5
4	Financial difficulties were experienced as a result of spending on healthcare	1	2	3	4	5

5	Over the past 12 months, you have experienced a significant delay in accessing medicine, specialists, equipment and diagnostic tests.	1	2	3	4	5
		**Strongly Disagree**	**Disagree**	**Unsure**	**Agree**	**Strongly Agree**
6	Over the past 12 months, I have experienced significant difficulty in accessing household items since markets have been closed due to lockdowns	1	2	3	4	5
7	The internet has taught me ways to learn new recipes/projects/ideas, when availability of resources was limited owing to the lockdowns	1	2	3	4	5
8	Over the past 12 months, financial transactions have become smoother, since most vendors have digital payment options	1	2	3	4	5
9	I experienced significant difficulty in adapting to new means of online payments	1	2	3	4	5

10	During the pandemic, I had limited options in accessing healthcare specialists due to poor connectivity	1	2	3	4	5
11	Having access and availability to good internet connection and technology- made adjusting to the pandemic easier	1	2	3	4	5
12	Over the past 12 months, having access to internet made me feel less socially isolated.	1	2	3	4	5
13	I feel like I lost a year of opportunities at work/college, due to limited access and availability of technology	1	2	3	4	5
14	The internet has strengthened the social community by letting strangers help each other, in times of need.	1	2	3	4	5
15	Due to limited access to internet and latest technology, I have struggled to be productive at work/college	1	2	3	4	5
16	Despite good access to internet and latest technology, I experienced significant difficulty in adapting to	1	2	3	4	5

	working/studying from home					

Section 3

In this section you are being asked questions related to your emotions and experiences during Covid 19.

S.no		Strongly Disagree	Disagree	Unsure	Agree	Strongly Agree
1	I felt tired, worn out, or exhausted during the past few weeks	1	2	3	4	5
2	The time I spend on family responsibilities predominantly interferes with my work responsibilities	1	2	3	4	5
3	Coronavirus (COVID-19) as an unusual circumstance has interfered with my work goals, and made it difficult to achieve them	1	2	3	4	5
4	I have been low in my motivation, to put efforts into my job.	1	2	3	4	5
5	It is not always easy for me to perform tasks on time	1	2	3	4	5
		**Strongly Disagree**	**Disagree**	**Unsure**	**Agree**	**Strongly Agree**
6	I feel I have lost interest in life.	1	2	3	4	5
7	Life seems meaningless after the covid experience.	1	2	3	4	5

8	I do not feel optimistic about life.	1	2	3	4	5
9	My motivation to do anything special has gone down.	1	2	3	4	5
10	I do not feel like working much now.	1	2	3	4	5
11	Doing anything new at work or at home does not excite me any longer.	1	2	3	4	5
12	The uncertainties about life are worrying me.	1	2	3	4	5
13	I cannot make sense of what’s going on in the world	1	2	3	4	5
14	Society has stopped making progress	1	2	3	4	5
15	People do not care about other people’s problems	1	2	3	4	5
16	My community is a source of comfort	1	2	3	4	5
17	This experience has made me worry about the future of my family members.	1	2	3	4	5
18	I am worried about the financial needs that may arise in future.	1	2	3	4	5
19	I am not optimistic about handling the needs that may arise in future.	1	2	3	4	5
20	I am sure that I can care about my family members in the future.	1	2	3	4	5

		Strongly Disagree	Disagree	Unsure	Agree	Strongly Agree
21	I felt cheerful most of the time	1	2	3	4	5
22	I was in good spirits most of the time	1	2	3	4	5
23	I was extremely happy most of the time	1	2	3	4	5
24	I was calm and peaceful most of the time	1	2	3	4	5
25	I was satisfied most of the time	1	2	3	4	5
		**Strongly Disagree**	**Disagree**	**Unsure**	**Agree**	**Strongly Agree**
26	If I were to have problems, I have people I could turn to	1	2	3	4	5
27	My family/ friends are very supportive of me	1	2	3	4	5
28	In difficult situations, I can manage my emotions	1	2	3	4	5
29	I can put up with my negative emotions	1	2	3	4	5
30	When faced with a problem I can usually find a solution	1	2	3	4	5
31	If I were in trouble, I know of others who would be able to help me	1	2	3	4	5
32	I can generally solve problems that occur	1	2	3	4	5
33	I can control my emotions	1	2	3	4	5

34	I can usually find a way of overcoming problems	1	2	3	4	5
35	I could find family of friends who listen to me if I needed them to	1	2	3	4	5
36	If faced with a set-back, I could probably find a way round the problem	1	2	3	4	5
37	I can handle my emotions	1	2	3	4	5
		**Strongly Disagree**	**Disagree**	**Unsure**	**Agree**	**Strongly Agree**
38	I've been turning to work or other activities to take my mind off things.	1	2	3	4	5
39	I've been concentrating my efforts on doing something about the situation I'm in.	1	2	3	4	5
40	I've been getting emotional support from others.	1	2	3	4	5
41	I've been doing something to think about it less, such as going to movies, watching TV, reading, daydreaming, sleeping, or shopping.	1	2	3	4	5
42	I've been accepting the reality of the fact that it has happened.	1	2	3	4	5
43	I've been learning to live with it.	1	2	3	4	5

44	I've been praying or meditating.	1	2	3	4	5
45	I've been saying things to let my unpleasant feeling escape.	1	2	3	4	5
46	I've been giving up the attempt to cope.	1	2	3	4	5
47	I've been trying to find comfort in my religion or spiritual beliefs	1	2	3	4	5
		**Strongly Disagree**	**Disagree**	**Unsure**	**Agree**	**Strongly Agree**
48	My experience with Covid-19 has prepared me to handle uncertainties in life.	1	2	3	4	5
49	I now make preparations for medical equipment and medicines in advance, to avoid shortage.	1	2	3	4	5
50	I think the public needs to continue being socially conscious, to avoid further impact.	1	2	3	4	5
51	I am worried economic progress is being affected because of covid-19 precautions.	1	2	3	4	5
52	I am worried about what would happen if other covid waves would come	1	2	3	4	5
53	The experience during these times has made me more anxious about future	1	2	3	4	5

54	I feel that I have will be more strong in future if such situations come.	1	2	3	4	5
55	I have learnt that we have to believe in God.	1	2	3	4	5
56	I have a strong sense of helplessness.	1	2	3	4	5
57	I can always manage to solve difficult problems if I try hard enough.	1	2	3	4	5
58	It is easy for me to stick to my aims and accomplish my goals.	1	2	3	4	5
59	If someone opposes me, I can find the means and ways to get what I want.	1	2	3	4	5
60	I am confident that I could deal efficiently with unexpected events.	1	2	3	4	5
61	Thanks to my resourcefulness, I know how to handle unforeseen situations.	1	2	3	4	5
62	I can solve most problems if I invest the necessary effort.	1	2	3	4	5
63	I can remain calm when facing difficulties	1	2	3	4	5

	because I can rely on my coping abilities.					
64	When I am confronted with a problem, I can usually find several solutions.	1	2	3	4	5
65	If I am in trouble, I can usually think of a solution	1	2	3	4	5
66	I can usually handle whatever comes my way.	1	2	3	4	5

Questionnaire in Hindi

प्रिय प्रतिभागियों,

हम देश के विभिन्न क्षेत्रों में रहने वाले लोगों पर कोविड के प्रभाव को समझने के लिए एक शोध परियोजना पर काम कर रहे हैं। आपकी प्रतिक्रियाओं से हमें यह जानने में मदद मिलेगी कि लोगों ने व्यक्तिगत और सामूहिक स्तर पर महामारी से संबंधित मुद्दों से कैसे निपटा। कोई सही या गलत उत्तर नहीं हैं। हमारे लिए आपकी प्रतिक्रियाएँ सही प्रतिक्रियाएँ हैं। आपसे कोई पहचान संबंधी जानकारी (जैसे, आपका नाम या पता) नहीं मांगी जाएगी। जानकारी पूरी तरह से गोपनीय है। हम आपको पूर्ण गोपनीयता का आश्वासन देते हैं। सर्वेक्षण में आपके मूल्यवान समय के 20 मिनट से अधिक नहीं लगेंगे। इस सर्वेक्षण में आपकी भागीदारी पूरी तरह से स्वैच्छिक है।

यदि आपके कोई प्रश्न हैं, तो कृपया misragirishwar@gmail.com, p.singh@hss.iitd.ac.in या madhumita.ramakrishna@uqidar.iitd.ac.in पर संपर्क करें।

कृपया इस सर्वेक्षण में भाग लेने के लिए अपनी सहमति दें। हाँ / नही-------------------

कृपया अपने बारे में कुछ जानकारी प्रदान करें। आपके द्वारा प्रदान की गई इस जानकारी का उपयोग केवल शोध उद्देश्य के लिए किया जाएगा।

आयु: ____

लिंग :______________________________________

रहने का स्थान: ☐मैट्रोपोलिटन सिटी ☐सिटी ☐शहर ☐ग्राम

वैवाहिक स्थिति ☐:विवाहित ☐अविवाहित ☐तलाकशुदा ☐विधवा

सामाजिक-आर्थिक स्तर ☐:निचला ☐ निचला मध्य ☐ऊपरी मध्य ☐ऊपरी

आपके परिवार की संरचना क्या है? कृपया उन पर टिक करें जो आप पर लागू होते हैं।

a. मैं अकेला रहता/रहती हूँ।

b. मैं अपने माता-पिता और / या जीवनसाथी के साथ रहता/रहती हूँ।

c. मैं एक संयुक्त-परिवार में रहता/रहती हूँ।

d. अन्य (कृपया निर्दिष्ट करें): __________

COVID-19 (कोरोनावायरस) के दौरान आप निम्न में से किसका अनुभव कर रहे हैं (या आपने अनुभव किया है)? (लागू होने वाले सभी की जाँच)। कृपया उन्हें रैंक भी दें, उस अनुभव को रैंक 1 दें जो सबसे प्रभावशाली और फिर अवरोही क्रम था।

___ COVID-19 का निदान किया जा रहा है

___कोविड-19 होने का डर

___किसी और को COVID-19 देने का डर

___ मित्रों, परिवार, भागीदारों आदि के बारे में चिंता करना।

___ अन्य लोगों से कलंक या भेदभाव (जैसे, आपकी पहचान, लक्षण होने या COVID-19 से संबंधित अन्य कारकों के कारण आपके साथ अलग व्यवहार करने वाले लोग)

___ व्यक्तिगत वित्तीय नुकसान (जैसे, खोई हुई मजदूरी, नौकरी छूटना, निवेश / सेवानिवृत्ति हानि, यात्रा से संबंधित रद्दीकरण)

___निराशा या ऊब

___ पर्याप्त बुनियादी आपूर्ति नहीं होना (जैसे, भोजन, पानी, दवाएं, रहने की जगह)

___अधिक चिंता

___अधिक अवसाद

___ अधिक नींद, कम नींद, या आपके सामान्य नींद पैटर्न में अन्य परिवर्तन

_शराब या अन्य पदार्थों का अधिक सेवन

___ अकेलापन

___ COVID-19 क्या है, इसे कैसे रोका जाए, या सोशल डिस्टेंसिंग/आइसोलेशन/क्वारंटाइन की आवश्यकता क्यों है, इस बारे में भ्रम

___ यह महसूस करना कि मैं खुद को या दूसरों को COVID-19 होने से रोककर अधिक से अधिक अच्छे में योगदान दे रहा था

___ परिवार, दोस्तों, भागीदारों, परामर्शदाता, या किसी और से भावनात्मक या सामाजिक समर्थन प्राप्त करना

___ परिवार, दोस्तों, भागीदारों, किसी संगठन, या किसी और से वित्तीय सहायता प्राप्त करना

___ अन्य कठिनाइयाँ या चुनौतियाँ

खंड 2

इस खंड में आपसे विभिन्न तकनीकों से संबंधित प्रश्न पूछे जा रहे हैं जिससे आपको महामारी के समय में अपना काम करने में मदद मिली। प्रत्येक कथन 5-पॉइंट रेटिंग स्केल से मेल खाता है (पूरी तरह से असहमत, असहमत, अनिश्चित, सहमत और दृढ़ता से सहमत)। यदि कथन आप पर लागू होता है, तो कृपया उपयुक्त रेटिंग पर निशान लगाएं।

**S.no**		**पूरी तरह से असहमत**	**असहमत**	**अनिश्चित**	**सहमत**	**पूरी तरह से सहमत**
1	पिछले 12 महीनों में आवश्यक स्वास्थ्य सेवाएं प्राप्त करना मेरे लिए आसान था	1	2	3	4	5
2	स्वास्थ्य देखभाल के संबंध में निम्नलिखित स्रोतों या चैनलों (स्कूल, काम, अस्पताल, टीवी) से जानकारी आसानी से उपलब्ध थी।	1	2	3	4	5
3	जरूरत पड़ने पर, मैं (वित्तीय रूप से) पहुंच कर लाभ पा सकता था:	1	2	3	4	5

	चिकित्सक, विशेषज्ञ, अस्पताल, दवाएं और चिकित्सा उपकरण					
4	स्वास्थ्य देखभाल पर खर्च के परिणामस्वरूप वित्तीय कठिनाइयों का अनुभव किया गया	1	2	3	4	5
5	पिछले 12 महीनों में, आपने दवा, विशेषज्ञ, उपकरण और नैदानिक परीक्षणों तक पहुँचने में काफी देरी का अनुभव किया है।	1	2	3	4	5
6	पिछले 12 महीनों में, मुझे घरेलू सामानों तक पहुँचने में काफी कठिनाई का सामना करना पड़ा है क्योंकि लॉकडाउन के कारण बाजार बंद हो गए हैं	1	2	3	4	5
7	इंटरनेट ने मुझे नए व्यंजनों/परियोजनाओं/विचारों को सीखने के तरीके सिखाए हैं, जब लॉकडाउन के कारण संसाधनों की उपलब्धता सीमित थी।	1	2	3	4	5
8	पिछले 12 महीनों में, वित्तीय लेनदेन आसान हो गए हैं, क्योंकि अधिकांश विक्रेताओं के पास डिजिटल भुगतान विकल्प हैं	1	2	3	4	5
9	मुझे ऑनलाइन भुगतान के नए साधनों को अपनाने में काफी कठिनाई का सामना करना पड़ा	1	2	3	4	5
10	महामारी के दौरान, खराब कनेक्टिविटी के कारण मेरे पास स्वास्थ्य विशेषज्ञों तक पहुंचने के सीमित विकल्प थे	1	2	3	4	5
11	अच्छे इंटरनेट कनेक्शन और तकनीक तक पहुंच और उपलब्धता होने से	1	2	3	4	5

	महामारी से तालमेल बिठाना आसान हो गया है					
12	पिछले 12 महीनों में, इंटरनेट तक पहुंच ने मुझे सामाजिक रूप से कम अलग-थलग महसूस कराया।	1	2	3	4	5
13	मुझे ऐसा लगता है कि मैंने काम/कॉलेज में सीमित पहुंच और प्रौद्योगिकी की उपलब्धता के कारण अवसरों का एक वर्ष खो दिया है	1	2	3	4	5
14	जरूरत के समय अजनबियों को एक-दूसरे की मदद करने की अनुमति देकर इंटरनेट ने सामाजिक समुदाय को मजबूत किया है।	1	2	3	4	5
15	इंटरनेट और नवीनतम तकनीक तक सीमित पहुंच के कारण, मुझे काम/कॉलेज में उत्पादक होने के लिए संघर्ष करना पड़ा	1	2	3	4	5
16	इंटरनेट और नवीनतम तकनीक तक अच्छी पहुंच के बावजूद, मुझे घर से काम करने/पढ़ाई करने में काफी कठिनाई का अनुभव हुआ	1	2	3	4	5

धारा 3

इस खंड में आपसे कोविड 19 के दौरान आपकी भावनाओं और अनुभवों से संबंधित प्रश्न पूछे जा रहे हैं।

**S.no**		**पूरी तरह से असहमत**	**असहमत**	**अनिश्चित**	**सहमत**	**पूरी तरह से सहमत**
1	पिछले कुछ हफ़्तों के दौरान मैं थका हुआ, घिसा-पिटा या थका हुआ महसूस कर रहा था	1	2	3	4	5
2	मैं पारिवारिक जिम्मेदारियों पर जो समय बिताता हूं वह	1	2	3	4	5

	मुख्य रूप से मेरी कार्य जिम्मेदारियों में हस्तक्षेप करता है					
3	एक असामान्य परिस्थिति के रूप में कोरोनावायरस (COVID-19) ने मेरे कार्य लक्ष्यों में हस्तक्षेप किया है, और उन्हें प्राप्त करना कठिन बना दिया है	1	2	3	4	5
4	मैं अपने काम में प्रयास करने के लिए अपनी प्रेरणा में कमी महसूस कर रहा हूं।	1	2	3	4	5
5	समय पर कार्य करना मेरे लिए हमेशा आसान नहीं होता है	1	2	3	4	5
6	मुझे लगता है कि मैंने जीवन में रुचि खो दी है।	1	2	3	4	5
7	कोविड के अनुभव के बाद जीवन बेमानी लगता है।	1	2	3	4	5
8	मैं जीवन के बारे में आशावादी महसूस नहीं करता।	1	2	3	4	5
9	कुछ खास करने की मेरी प्रेरणा कम हो गई है।	1	2	3	4	5
10	अब ज्यादा काम करने का मन नहीं करता।	1	2	3	4	5
11	काम पर या घर पर कुछ भी नया करना मुझे अब उत्साहित नहीं करता है।	1	2	3	4	5
12	जीवन को लेकर अनिश्चितताएं मुझे चिंतित कर रही हैं।	1	2	3	4	5

13	मैं समझ नहीं पा रहा हूं कि दुनिया में क्या हो रहा है	1	2	3	4	5
14	समाज ने प्रगति करना बंद कर दिया है	1	2	3	4	5
15	लोगों को दूसरे लोगों की समस्याओं की परवाह नहीं है	1	2	3	4	5
16	मेरा समुदाय आराम का स्रोत है	1	2	3	4	5
17	इस अनुभव ने मुझे अपने परिवार के सदस्यों के भविष्य के बारे में चिंतित कर दिया है।	1	2	3	4	5
18	मैं भविष्य में उत्पन्न होने वाली वित्तीय जरूरतों को लेकर चिंतित हूं।	1	2	3	4	5
19	मैं भविष्य में उत्पन्न होने वाली जरूरतों को संभालने के बारे में आशावादी नहीं हूं।	1	2	3	4	5
20	मुझे यकीन है कि मैं भविष्य में अपने परिवार के सदस्यों की देखभाल कर सकता हूं।	1	2	3	4	5
21	मैं ज्यादातर समय खुशनुमा महसूस करता था	1	2	3	4	5
22	मैं ज्यादातर समय अच्छी आत्माओं में था	1	2	3	4	5
23	मैं ज्यादातर समय बेहद खुश रहता था	1	2	3	4	5
24	मैं ज्यादातर समय शांत और शांत रहता था	1	2	3	4	5
25	मैं ज्यादातर समय संतुष्ट रहता था	1	2	3	4	5

26	अगर मुझे समस्याएँ होतीं, तो मेरे पास ऐसे लोग होते जिन्हें मैं बदल सकता था	1	2	3	4	5
27	मेरा परिवार/दोस्त मुझे बहुत सपोर्ट करते हैं	1	2	3	4	5
28	कठिन परिस्थितियों में, मैं अपनी भावनाओं को प्रबंधित कर सकता हूं	1	2	3	4	5
29	मैं अपनी नकारात्मक भावनाओं को सह सकता हूं	1	2	3	4	5
30	जब किसी समस्या का सामना करना पड़ता है तो मैं आमतौर पर समाधान ढूंढ सकता हूं	1	2	3	4	5
31	अगर मैं मुसीबत में होता, तो मैं दूसरों को जानता हूं जो मेरी मदद करने में सक्षम होंगे	1	2	3	4	5
32	मैं आम तौर पर होने वाली समस्याओं को हल कर सकता हूं	1	2	3	4	5
33	मैं अपनी भावनाओं को नियंत्रित कर सकता हूं	1	2	3	4	5
34	मैं आमतौर पर समस्याओं पर काबू पाने का एक तरीका ढूंढ सकता हूं	1	2	3	4	5
35	मुझे ऐसे दोस्तों का परिवार मिल सकता है जो मेरी जरूरत पड़ने पर मेरी बात सुनते हैं	1	2	3	4	5
36	अगर मुझे असफलता का सामना करना पड़ता है, तो	1	2	3	4	5

	शायद मैं समस्या का समाधान ढूंढ सकता हूं					
37	मैं अपनी भावनाओं को संभाल सकता हूं	1	2	3	4	5
38	मैं चीजों से अपना ध्यान हटाने के लिए काम या अन्य गतिविधियों की ओर रुख कर रहा हूं।	1	2	3	4	5
39	मैं जिस स्थिति में हूं, उसके बारे में कुछ करने पर अपने प्रयासों पर ध्यान केंद्रित कर रहा हूं।	1	2	3	4	5
40	मुझे दूसरों से भावनात्मक समर्थन मिल रहा है।	1	2	3	4	5
41	मैं इसके बारे में कम सोचने के लिए कुछ कर रहा हूं, जैसे कि फिल्में देखना, टीवी देखना, पढ़ना, दिवास्वप्न देखना, सोना या खरीदारी करना।	1	2	3	4	5
42	मैं इस तथ्य की वास्तविकता को स्वीकार कर रहा हूं कि यह हुआ है।	1	2	3	4	5
43	मैं इसके साथ रहना सीख रहा हूं।	1	2	3	4	5
44	मैं प्रार्थना कर रहा हूं या ध्यान कर रहा हूं।	1	2	3	4	5
45	मैं अपनी अप्रिय भावना से बचने के लिए चीजें कह रहा हूं।	1	2	3	4	5
46	मैं सामना करने का प्रयास छोड़ रहा हूं।	1	2	3	4	5

47	मैं अपने धर्म या आध्यात्मिक विश्वासों में आराम पाने की कोशिश कर रहा हूं	1	2	3	4	5
48	कोविड-19 के साथ मेरे अनुभव ने मुझे जीवन में अनिश्चितताओं से निपटने के लिए तैयार किया है।	1	2	3	4	5
49	मैं अब चिकित्सा उपकरणों और दवाओं की कमी से बचने के लिए पहले से तैयारी करता हूं।	1	2	3	4	5
50	मुझे लगता है कि आगे प्रभाव से बचने के लिए जनता को सामाजिक रूप से जागरूक रहने की जरूरत है।	1	2	3	4	5
51	मैं चिंतित हूं कि कोविड-19 सावधानियों के कारण आर्थिक प्रगति प्रभावित हो रही है।	1	2	3	4	5
52	मुझे इस बात की चिंता है कि अगर अन्य कोविड लहरें आएंगी तो क्या होगा?	1	2	3	4	5
53	इन समयों के अनुभव ने मुझे भविष्य के बारे में और अधिक चिंतित कर दिया है	1	2	3	4	5
54	मुझे लगता है कि अगर भविष्य में ऐसी स्थितियां आती हैं तो मैं और अधिक मजबूत हो जाऊंगा।	1	2	3	4	5

55	मैंने सीखा है कि हमें भगवान में विश्वास करना है।	1	2	3	4	5
56	मुझमें लाचारी की प्रबल भावना है।	1	2	3	4	5
57	मैं हमेशा कठिन समस्याओं को हल करने का प्रबंधन कर सकता हूं यदि मैं पर्याप्त प्रयास करता हूं।	1	2	3	4	5
58	मेरे लिए अपने लक्ष्य पर टिके रहना और अपने लक्ष्यों को पूरा करना आसान है।	1	2	3	4	5
59	अगर कोई मेरा विरोध करता है, तो मैं जो चाहता हूं उसे पाने के साधन और तरीके ढूंढ सकता हूं।	1	2	3	4	5
60	मुझे विश्वास है कि मैं अप्रत्याशित घटनाओं से कुशलता से निपट सकता हूं।	1	2	3	4	5
61	मेरी संसाधन कुशलता के लिए धन्यवाद, मैं जानता हूं कि अप्रत्याशित परिस्थितियों को कैसे संभालना है।	1	2	3	4	5
62	यदि मैं आवश्यक प्रयास करता हूँ तो मैं अधिकांश समस्याओं का समाधान कर सकता हूँ।	1	2	3	4	5

63	कठिनाइयों का सामना करते समय मैं शांत रह सकता हूं क्योंकि मैं अपनी मुकाबला करने की क्षमताओं पर भरोसा कर सकता हूं।	1	2	3	4	5
64	जब मैं किसी समस्या का सामना करता हूं, तो मैं आमतौर पर कई समाधान ढूंढ सकता हूं।	1	2	3	4	5
65	अगर मैं मुसीबत में हूं, तो मैं आमतौर पर समाधान के बारे में सोच सकता हूं	1	2	3	4	5
66	मैं आमतौर पर जो कुछ भी मेरे रास्ते में आता है उसे संभाल सकता हूं।	1	2	3	4	5

TABLES

Table 1: Table 1: Split-Half and Cronbach Alpha Values for Measures in the Questionnaire

Measures	Guttman's Lambda 4	Cronbach Alpha
Impact of Technology	0.88	0.77
Motivation to Work	0.85	0.82
Emotional Well-Being	0.86	0.85
Resilience	0.94	0.9
Self-Efficacy	0.92	0.88
